# Genome-Wide Analysis and Expression Profiling of *DUF668* Genes in *Glycine max* under Salt Stress

**DOI:** 10.3390/plants12162923

**Published:** 2023-08-11

**Authors:** Madiha Zaynab, Yasir Sharif, Zhaoshi Xu, Sajid Fiaz, Rashid Al-Yahyai, Hamad. A. Yadikar, Najla Amin T. Al Kashgry, Sameer H. Qari, Monther Sadder, Shuangfei Li

**Affiliations:** 1 College of Physics and Optoelectronic Engineering, Shenzhen University, Shenzhen 518060, China; madiha.zaynab14@gmail.com; 2 Shenzhen Key Laboratory of Marine Bioresource & Eco-Environmental Sciences, College of Life Sciences and Oceanography, Shenzhen University, Shenzhen 518060, China; 3 College of Plant Protection, Fujian Agriculture and Forestry University, Fuzhou 350002, China; 4 Institute of Crop Science, Chinese Academy of Agricultural Sciences, National Key Facility for Crop Gene Resources and Genetic Improvement, Key Laboratory of Biology and Genetic Improvement of Triticeae Crops, Ministry of Agriculture, Beijing 100081, China; 5 Department of Plant Breeding and Genetics, The University of Haripur, Haripur 22620, Pakistan; 6 Department of Plant Sciences, College of Agricultural and Marine Sciences, Sultan Qaboos University, Al-Khod, P.O. Box 34, Muscat 123, Oman; 7 Department of Biological Sciences, Faculty of Science, Kuwait University, P.O. Box 5969, Safat 13060, Kuwait; 8 Department of Biology, College of Science, Taif University, P.O. Box 11099, Taif 21944, Saudi Arabia; 9 Department of Biology, Al-Jumum University College, Umm Al-Qura University, Makkah 21955, Saudi Arabia; 10 School of Agriculture, University of Jordan, Amman 11942, Jordan

**Keywords:** phytohormones, miRNAs, gene expression, abiotic stress, phylogeny, duplication

## Abstract

The *DUF668* gene performs a critical role in mitigating the impact of abiotic stress factors. In this study, we identified 30 *DUF668* genes in a soybean genome, distributed across fifteen chromosomes. The phylogenetic analysis classified the *DUF668* genes into three groups (group I, group II, and group III). Interestingly, gene structure analysis illustrated that several *GmDUF668* genes were without introns. Furthermore, the subcellular localization results suggested that GmDUF668 proteins were present in the nucleus, mitochondria, cytoplasm, and plasma membrane. *GmDUF668* promoters were analyzed in silico to gain insight into the presence of regulatory sequences for TFs binding. The expression profiling illustrated that *GmDUF668* genes showed expression in leaves, roots, nodules, and flowers. To investigate their response to salt stress, we utilized the RNA sequencing data of *GmDUF668* genes. The results unveiled that *GmDUF668-8*, *GmDUF668-20*, and *GmDUF668-30* genes were upregulated against salt stress treatment. We further validated these findings using qRT-PCR analysis. These findings provide a scientific basis to explore the functions of *GmDUF668* genes against different stress conditions.

## 1. Introduction

Environmental imbalances potentially cause up to 70% of an annual yield loss [1], resulting in substantial agricultural losses worldwide due to abiotic stress factors affecting plant development [2]. Among the factors affecting crop growth, salt is considered a primary factor [3]. Generally, plants lack the structures that directly perform a role in environmental perception, but they are capable of responding to environmental change [4]. Plants possess defense mechanisms at cellular and molecular levels to oppose cell damage against stresses [5]. The genome data availability helps the researcher to identify and characterize the stress-responsive gene families such as FKBP, MAPK, NAC, PEPC, bHLH, Aux/IAA, bZIP, TIFY, HSF, and GRAS transcription factor [6]. Within the plant genomes, numerous stress-related proteins with highly conserved domains perform critical functions in various biological processes under stress [6]. Thus, the genes with these hypothetical domains have been categorized as domains of unknown functions (DUF) [6]. The Pfam database currently contains 18,259 gene members, of which almost 5645 genes are members of the DUF families [7]. Several DUF families, such as *DUF581* and *DUF724*, were identified in *A. thaliana*, and *DUF1618*, *DUF936*, *DUF866*, *DUF810*, and *DUF221* in rice.

Different *DUF* gene members have a role against abiotic stress, such as *OsDUF810.7* (DUF2275) [8], DUF966 (*OsDSR2*) [9], and DUF1644 (*SIDP361*) [10]. Previous research on *A. thaliana* revealed that *AT3g55990* (DUF231) is a negative regulator against the acclimation of cold [11]. Another study reported that it also ceased the gene expression of the *ARTDUF1/ARTDUF2* (RING-DUF1117 E3 ubiquitin ligase) [12]. Similarly, the *OsSIDP366* (DUF1644) was identified as a regulator in mitigating salt and drought stresses in rice [13]. Overexpression of the *OsSIDP366* gene resulted in enhanced resistance against salt and drought stresses in transgenic rice [10]. The DUF283 domain was needed in siRNA processing during gene silencing [14], while the *DUF538* gene family members have chlorophyll hydrolyzing abilities [15]. The overexpression of the salt-induced gene *TaSRHP* (DUF581 domain containing) increased the resistance in response to drought and salt stress factors [16]. The stress tolerance of *DUF668* genes has been studied in model plants. The current study illustrated that the *DUF668* gene family plays essential roles against abiotic stress factors in soybean.

*Glycine max* L (soybean) is an economically important oil and protein crop [17], but its productivity and growth are badly affected by salinity. The genome sequencing of soybean was performed ten years ago but still lacks information related to the *DUF668* gene family. To bridge this gap, our study identified 30 *GmDUF668* genes in the soybean genome, and their phylogenetic relations, gene structure, promoters, and expression analysis in various tissues were studied. Specifically, we examined the *GmDUF668* gene expression under salt stress. The expression levels of *GmDUF668-8* and *GmDUF668-20* genes were studied using qRT-PCR after salinity treatment. Our findings provide a scientific basis to explore the role of the *GmDUF668s* against different stress conditions.

## 2. Material and Methods

### 2.1. DUF668 Genes Identification

This study used the BLASTP search and HMM (Hidden Markov Model) methods to identify *DUF688* genes in the soybean genome [18]. For the BLASTP search, we used AtDUF668 amino acid sequences as a query. The AtDUF668 amino acid sequences were obtained from the TAIR database (https://www.arabidopsis.org/ (accessed on 11 December 2022) [19]. The HMMER 3.13 [20] tool was used to find the *DUF668* genes. The HMM files of the DUF668 domain (PF05003) were obtained from the Pfam database [21]. Finally, the sequences containing the PF05003 domain were taken as *DUF668* gene members, and 30 *GmDUF668* genes were identified in the *G. max* genome. The *Brachypodium distachyon* and *Sorghum bicolor* DUF668 protein sequences were obtained from the Phytozome JGI 12.0 dataset (http://phytozome.jgi.doe.gov/pz/portal.html (accessed on 11 December 2022) [22]. The information on all identified *DUF668* gene members is mentioned in the supplementary data Table S2.

Physiochemical properties of the GmDUF668 proteins were obtained using the ExPASy server [23]. The GmDUF668 protein’s subcellular localizations were predicted using CELLO v.2.57 [24]. Intron-exon configurations of all *GmDUF668* genes were estimated using TBtools [25]. Moreover, the *GmDUF668* gene’s conserved motifs were obtained through the MEME tool [26]. The distribution of motifs was estimated through TBtools software.

### 2.2. Phylogenetic Relationships, Chromosomal Location, and Synteny Analysis

A phylogenetic tree among *S. Bicolour (SbDUF668)*, *A. thaliana (AtDUF668)*, *B. distachyon (BdDUF668)*, and *G. max (GmDUF668)* was created to identify their evolutionary links. DUF668 protein sequences were aligned by Clustal alignment, and a tree was generated by MEGA X software using the neighbor-joining method with 1000 bootstrap replicates [27]. The phylogenetic tree was beautified by iTOL [28]. The chromosomal location data associated with *GmDUF668s* were attained from the general feature format (GFF) file of the soybean genome [18], while the figure was generated using TBtools. The synteny analysis was performed using the Circoletto web tool (http://bat.ina.certh.gr/tools/circoletto/ (accessed on 11 December 2022) [29]. Furthermore, the ratios of Ka/Ks were estimated through KaKs 2.0 Calculator (https://sourceforge.net/projects/kakscalculator2/ (accessed on 11 December 2022). The expected divergence time (t) for the duplicated gene pairs was calculated through t = Ks/2r with r = 6.161029 × 10^−9^ [30].

### 2.3. Cis-Regulatory Elements Prediction in the GmDUF668 Promoters

The upstream 2 kb sequences of *GmDUF668* genes were extracted from the soybean genome to forecast the *cis*-regulatory factors in *GmDUF668* promoters. All *GmDUF668s* promoter sequences were submitted to the PlantCARE site [31] and figured out with TBtools.

### 2.4. Prediction of miRNAs Targeting GmDUF668 Genes

The *GmDUF668* CDS sequences were used to predict the miRNAs using the psRNATarget site [32]. In addition, the Cytoscape tool was used to generate the interaction network of *GmDUF668s* and miRNAs [33].

### 2.5. Expression Profiling of GmDUF668 Genes

A publicly available database was used to study *GmDUF668s* expression in various tissues. For tissue expression analysis, transcriptome data were derived from the publicly existing database Atlas of soybeans (http://bar.utoronto.ca/eplant_soybean/ (accessed on 11 December 2022)) [34]. Supplementary Table S6a provides information on the expression of *GmDUF668* genes in roots, nodules, leaves, and flowers. The *GmDUF668s* expression was observed against salt stress conditions (1 h versus control = 0 h; 12 h versus control = 0 h). In addition, *GmDUF668s* expression data under abiotic stress were obtained from GEO series accession number GSE57252. Moreover, the abundance of the transcripts was estimated as FPKM (fragment per kilobase million). An expression heat map was created through the TBtools program [25].

### 2.6. Plant Materials and Stress Treatments

Soybean variety Williams 82 was used to study the gene expression in this research. Seeds of Williams 82 were grown in a greenhouse in a mixture of humus and vermiculite (humus: vermiculite  =  1:1) until the seedlings grew to 10 cm and had two new leaves, and then the entire group of seedlings was subjected to salt stress. For NaCl treatment, the seedlings were treated with 250 mM NaCl, and the leave samples were taken at 0 h, 1 h, 2 h, 4 h, 8 h, 6 h, 12 h, 24 h, and 48 h. Later, leaves were transferred to the liquid nitrogen and stored at −80 °C [35].

### 2.7. RNA Extraction and qRT-PCR

The total RNA was extracted from samples using the Trizol reagent (TIANGEN, Beijing, China). The cDNA was synthesized through a PrimeScriptTM RT Reagent Kit (Takara, Shiga, Japan) [36]. Primer Premier 5.0 was used to design the primers. Soybean *Actin* was used as an internal control. Three biological replicates were used for qRT-PCR [37]. All designed primers are mentioned in Supplementary Table S1.

### 2.8. Data Analysis

The relative expression levels using qRT-PCR analysis were calculated through the 2^−∆∆CT^ method. Statistical significance was calculated by ANOVA (Analysis of Variance) and LSD (least standard deviation) multiple comparison tests (*p* ≤ 0.05). Moreover, the graphs were drawn by GraphPad Prism 9. The mean values are ±SE (n = 3). Different numbers represent the differences between expression levels at different time points.

## 3. Results

### 3.1. Comprehensive Characterization of GmDUF668s

**Figure 1:**
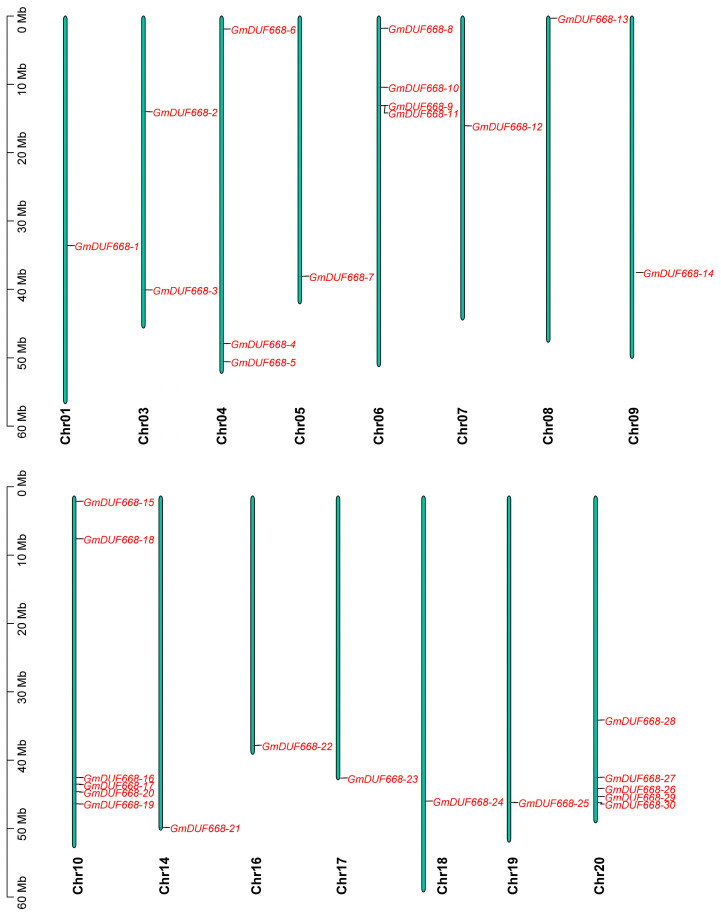
Locations of *GmDUF668s* on chromosomes. The chromosomes are shown by the green color; the genes by the red color.

In this research, 30 *DUF668* genes were discovered in the *Glycine max* genome (Table 1; Supplementary Table S2). The maximum *GmDUF668* gene numbers (6) were mapped on Chr10. Subsequently, Chr20 contained five genes, Chr06 contained four genes, Chr04 had three genes, and Chr03 contained two genes. The lowest number of the *GmDUF668* gene (1) was mapped on Chr01, Chr05, Chr08, Chr09, Chr14, Chr16, Chr17, Chr18, and Chr19 (Figure 1). Comprehensive detail of all 30 *GmDUF668* genes is mentioned in Table 1. In brief, the amino acid length ranged from 435 (*GmDUF668-14*) to 659 (*GmDUF668-19*). The exon numbers were found from one (*GmDUF668-1*, *GmDUF668-2*, *GmDUF668-3*, *GmDUF668-5*, *GmDUF668-10*, *GmDUF668-12*, *GmDUF668-14*, *GmDUF668-15*, *GmDUF668-16*, *GmDUF668-17*, *GmDUF668-18*, *GmDUF668-20*, *GmDUF668-22*, *GmDUF668-25*, *GmDUF668-26*, *GmDUF668-28*, and *GmDUF668-30*) to 12 (*GmDUF668-4*, *GmDUF668-6*, *GmDUF668-7*, *GmDUF668-8*, *GmDUF668-9*, *GmDUF668-11*, *GmDUF668-13*, *GmDUF668-19*, *GmDUF668-21*, *GmDUF668-23*, and *GmDUF668-27*) (Table 1). Genes *GmDUF668-4*, *GmDUF668-6*, *GmDUF668-7*, *GmDUF668-8*, *GmDUF668-9*, *GmDUF668-11*, *GmDUF668-13*, *GmDUF668-19*, *GmDUF668-21*, *GmDUF668-23*, and *GmDUF668-27* comprised the maximum introns number (11), while the genes *GmDUF668-1*, *GmDUF668-2*, *GmDUF668-3*, *GmDUF668-5*, *GmDUF668-10*, *GmDUF668-12*, *GmDUF668-14*, *GmDUF668-15*, *GmDUF668-16*, *GmDUF668-17*, *GmDUF668-18*, *GmDUF668-20*, *GmDUF668-22*, *GmDUF668-25*, *GmDUF668-26*, *GmDUF668-28*, and *GmDUF668-30* were found without introns (Table 1). The MW of the GmDUF668 proteins ranged from 48.92 kDa (GmDUF668-14) to 73.61 kDa (GmDUF668-19). The pI (isoelectric point) ranged from 6.77 (GmDUF668-29) to 9.59 (GmDUF668-18). The results of subcellular localization revealed that only one GmDUF668 protein was located in the extracellular matrix. Two GmDUF668 proteins were located on the plasma membrane, three GmDUF668 proteins were located in the cytoplasm, nine in the mitochondria, and fifteen GmDUF668 proteins were found in the nucleus (Table 1). Particularly, some GmDUF668 proteins were situated in more than one site (Table 1). Conversely, six genes in *A. thaliana (AtDUF668-1-AtDUF668-6*), 11 genes in *B. distachyon (BdDUF668-1-BdDUF668-11)*, and 12 genes in the *S. bicolor* (*SbDUF668-1-SbDUF668-12*) genome were identified.

### 3.2. Phylogenetic Relation of the DUF668s

**Figure 2:**
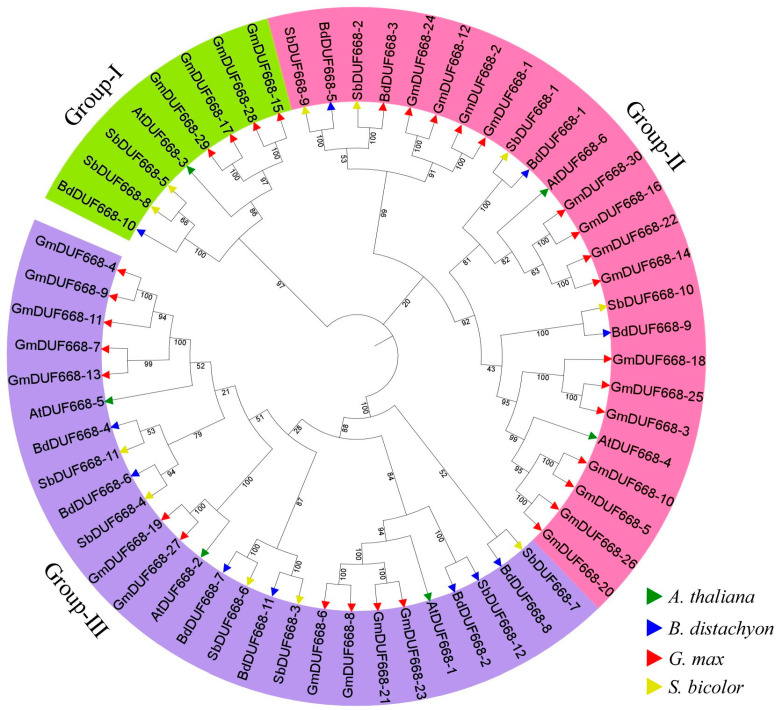
Phylogenetic tree among *A. thaliana*, *B*. *distachyon*, *S. bicolor*, and *G. max*. Overall, 6 *AtDUF668* from *A. thaliana* (green), 11 *BdDUF668* from *B*. *distachyon* (blue), 12 *SbDUF668* from *S. bicolor* (yellow), and 30 *GmDUF668* from *G. max* (red) were used.

A phylogenetic tree was generated to observe the evolutionary relationships among *BdDUF668*, *SbDUF668*, *AtDUF668*, and *GmDUF668* genes (Figure 2; Supplementary Table S2). The alignment was performed using DUF668 protein sequences of the *S. bicolor*, *B. distachyon*, *A. thaliana*, and *G. max*. In the present study results, *DUF668* genes from *A. thaliana*, *S. bicolor*, *B. distachyon*, and *G. max* were categorized into three main groups (I, II, and III), and the *GmDUF668s* of the different species were present in all groups. Group I comprised eight *DUF668* members (one *AtDUF668*, one *BdDUF668*, two *SbDUF668*, and four *GmDUF668*), group II comprised 25 *DUF668* members (two *AtDUF668*, four *BdDUF668*, four *SbDUF668*, and 15 *GmDUF668*). Further, group III contained 26 *DUF668* members (three *AtDUF668*, six *BdDUF668*, six *SbDUF668*, and 11 *GmDUF668*) (Figure 2; Supplementary Table S2). The clustering of *DUF668* genes within the same group suggested the possibility that they would perform similar tasks. Group III mainly contained more DUF668 (26) members than other groups (Figure 2). Additionally, group II showed a higher abundance of of *GmDUF668s* (Figure 2).

### 3.3. Analysis of the Cis-Elements in GmDUF668 Genes Promoter Sites

**Figure 3:**
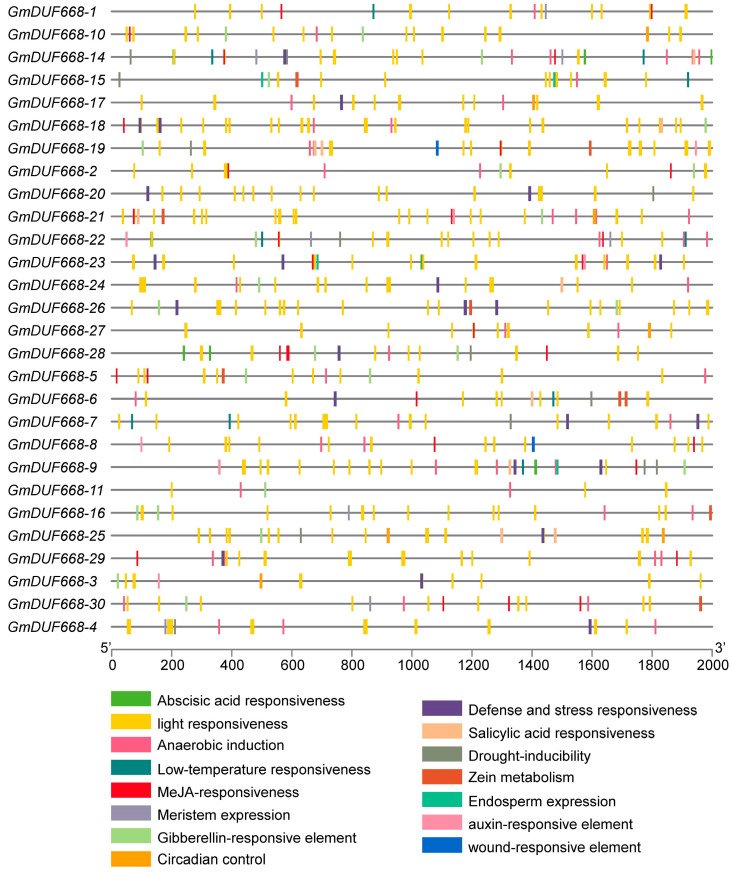
*Cis*-regulatory elements of the *GmDUF668s*. Different color boxes show different identified elements. Phytohormones-responsive, development- and growth-responsive, and abiotic stress-responsive elements.

To understand the *GmDUF668* promoter’s regulatory role toward soybean development and their ability to tolerate abiotic stress, promoter *cis*-regulatory factors were investigated. The details of *cis*-acting elements are mentioned in Supplementary Table S4. The analysis revealed three groups of *cis*-acting elements: phytohormones-responsive, development- and growth-responsive, and abiotic stress-responsive elements (Figure 3; Supplementary Table S4). This study observed six abiotic stress-related elements (light, drought, defense and stress, wounds, low temperature, and anaerobic condition). Moreover, these elements contain GTI-motif, I-box, GA-motif, Box 4, and ATCT-motif. Similarly, phytohormone-responsive elements include ABA, IAA, MeJA, GA, and SA (contain CGTCA-motif/TGACG-motif, GARE-motif/TATC-box, P-box, AuxRR-core/TGA-box/TGA-element) ABRE, TCA-element, and SARE. These findings suggest that genes specific to these elements play a crucial role in understanding their defensive function against hormone treatments. However, this study identified four development and growth-related elements: circadian control, endosperm expression, meristem expression, and zein metabolism. The development- and growth-related elements consist of GCN4-motif/AACA-motif, O2-site, circadian, and CAT-box (Figure 3; Supplementary Table S4), indicating their role during different developmental and growth stages in the soybean plant. This study also reported that *GmDUF668* genes expression profiles might fluctuate during developmental stages in response to abiotic and phytohormonal stresses.

### 3.4. Conserved Motifs and Gene Structure Analysis of GmDUF668 Genes

**Figure 4:**
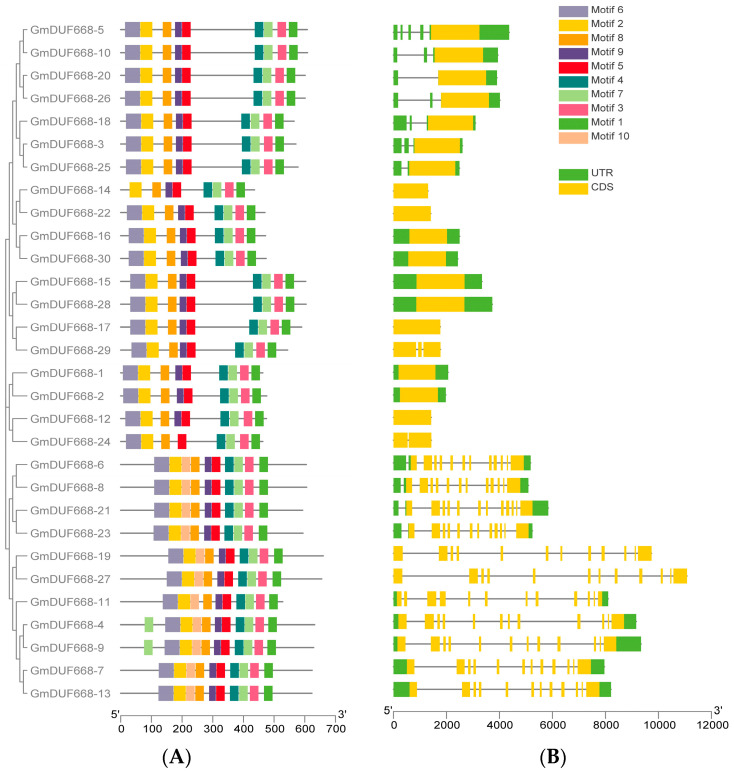
Motifs and gene structure of GmDUF668s; (**A**) *GmDUF668s* conserved motifs. Different motifs are represented by different colors. (**B**) *GmDUF668* gene’s structure. Exons are denoted by the yellow color, green shows UTR, and the black horizontal lines show introns.

The arrangement of introns-exons and their conserved motifs were studied to understand the *DUF668s’* advancement in the soybean genome. The findings of this research showed that the exon numbers varied from 1–12, while introns varied from 0–11 (Figure 4; Supplementary Table S3). In brief, seventeen genes contain one exon and zero introns; one gene consists of one intron and two exons; one gene contains two introns and three exons, while eleven genes contain twelve exons and eleven introns (Figure 4; Supplementary Table S3). Moreover, *GmDUF668s* belonging to the same group had the same structure (Figure 4). Of all genes, *GmDUF668-27* had the longest sequence; only some genes showed an intricate structure, including *GmDUF668-19*, *GmDUF668-6*, *GmDUF668-8*, *GmDUF668-21*, *GmDUF668-23*, *GmDUF668-4*, *GmDUF668-9*, and *GmDUF668-7* (Figure 4). These outcomes suggested that *DUF668s* maintained a consistent intron-exon composition during the soybean genome evolution, with *GmDUF668s* within the same group containing remarkably parallel gene structures.

**Figure 5:**
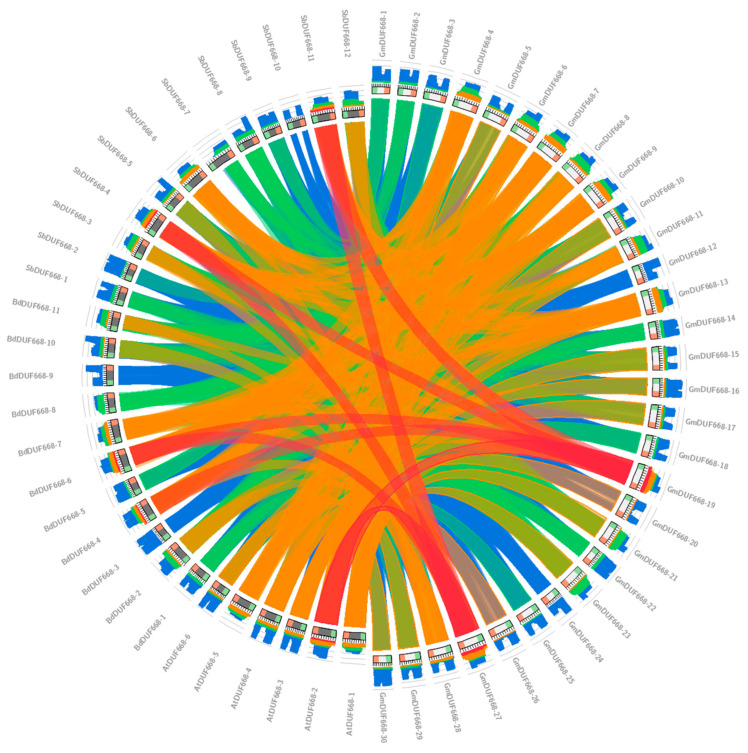
The comparative synteny analysis of *DUF668s* among *S.bicolor*, *G. max*, *B. distachyon*, and *A. thaliana*.

A total of ten conserved motifs were predicted, and their complete dataset is given in Supplementary Table S3. Motif distribution was identical within the group (Figure 4), but a few motifs were found in some genes and were absent in others. For example, a few genes, including *GmDUF668-14*, were limited to eight motifs. *GmDUF668-6*, *GmDUF668-8*, *GmDUF668-21*, *GmDUF668-23*, *GmDUF668-19*, *GmDUF668-27*, *GmDUF668-11*, *GmDUF668-7*, *GmDUF668-13*, *GmDUF668-4*, and *GmDUF668-9* contained ten motifs (Figure 4). Further, *GmDUF668-5*, *GmDUF668-10*, *GmDUF668-20*, *GmDUF668-26*, *GmDUF668-18*, *GmDUF668-3*, and *GmDUF668-25* had nine motifs (Figure 5). In brief, the gene organization consistency in the groups was realistically invariable with appraising conserved gene structure, phylogenetic relation, and motif structure representing DUF668 proteins containing extremely well-sustained amino acids.

### 3.5. Gene Duplication and Synteny Analysis of DUF668 Genes

Gene duplication supports the evolution and expansion of plant gene families. The gene duplication analysis results reported four *GmDUF668* gene pairs unevenly distributed on the chromosomes. Primarily, Chr10 and Chr20 housed two *GmDUF668* gene pairs (*GmDUF668-19: GmDUF668-27* and *GmDUF668-17: GmDUF668-29*), while one *GmDUF668* gene pair (*GmDUF668-12: GmDUF668-24*) was housed by Chr07 and Chr18, and one (*GmDUF668-14: GmDUF668-22*) was housed by Chr09 and Chr14. The outcomes illustrated that the segmental duplication participated in the *GmDUF668* gene expansion of the soybean genome (Table 2). Remarkably, segmentally duplicated gene pairs were observed in this study. In addition, the Ka/Ks value was calculated for each duplicated gene pair to estimate their evolutionary rate (Table 2). The Ka/Ks value was predictably used as selection pressure. If Ka/Ks > 1, it was considered positive selection pressure; when Ka/Ks = 1, it was considered neutral selection, and the Ka/Ks < 1 value was considered purifying selection [38]. These results indicated that most *GmDUF668* duplicated gene pairs experienced purifying selection during duplication (Table 2). Moreover, the divergence time varied from 8.83 million years to 22.54 million years for duplicated gene pairs *GmDUF599-19/GmDUF599-27* and *GmDUF599-12/GmDUF599-24*, respectively. The comparative synteny analysis of *DUF668s* was performed in *S. bicolor*, *G. max*, *B. distachyon*, and *A. thaliana*. The synteny diagram represents a significant relation among these species (Figure 5). Furthermore, it was identified that the soybean *GmDUF668-4* gene sequence showed synteny with the *S. bicolor SbDUF668-6* gene sequence, while the soybean *GmDUF668-19* gene sequence illustrated synteny with the *SbDUF668-11* gene of *S. bicolor* (Figure 5). Furthermore, the soybean *GmDUF668-19* gene sequence also showed synteny with *BdDUF668-4* and *BdDUF668-6* genes’ sequences of *B. distachyon* (Figure 5). The *GmDUF668-27* gene sequence also showed synteny with *S. bicolor SbDUF668-4* and *SbDUF668-11* genes’ sequences. In contrast, the *GmDUF668-19* gene showed synteny with *AtDUF668-2* gene sequences (Figure 5).

### 3.6. Genome-Wide Investigation of miRNAs Targeting GmDUF668 Genes

**Figure 6:**
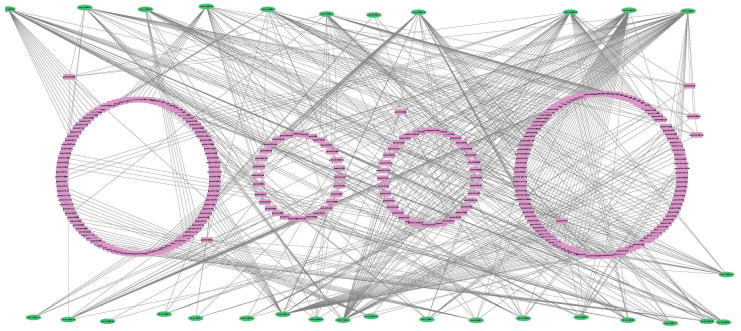
Predicted miRNAs targeting *GmDUF668* genes. Overall, 135 miRNAs belonging to 73 different families targeted 22 *GmDUF668* genes.

To understand the miRNA-mediated, post-transcriptional modifications, we analyzed the miRNAs targeting *GmDUF668* genes (Figure 6 and Supplementary Table S5). Identified miRNAs were representatives of various families and exhibited different targeting patterns. The complete data of targeted sites of miRNAs are mentioned in Supplementary Table S5. Analysis outcomes reported that gma-miR167 targeted five genes (*GmDUF668-15*, *GmDUF668-16*, *GmDUF668-18*, *GmDUF668-20*, and *GmDUF668-26*). Four gma-miR397 members targeted three genes (*GmDUF668-3*, *GmDUF668-9*, and *GmDUF668-27*); forty-nine miRNAs targeted seven genes (*GmDUF668-3*, *GmDUF668-5*, *GmDUF668-6*, *GmDUF668-10*, *GmDUF668-13*, *GmDUF668-15*, and *GmDUF668-28*); twenty-four gma-miR395 members targeted two genes (*GmDUF668-21* and *GmDUF668-23*) (Figure 6).

### 3.7. GmDUF668s Expression in Different Tissues

**Figure 7:**
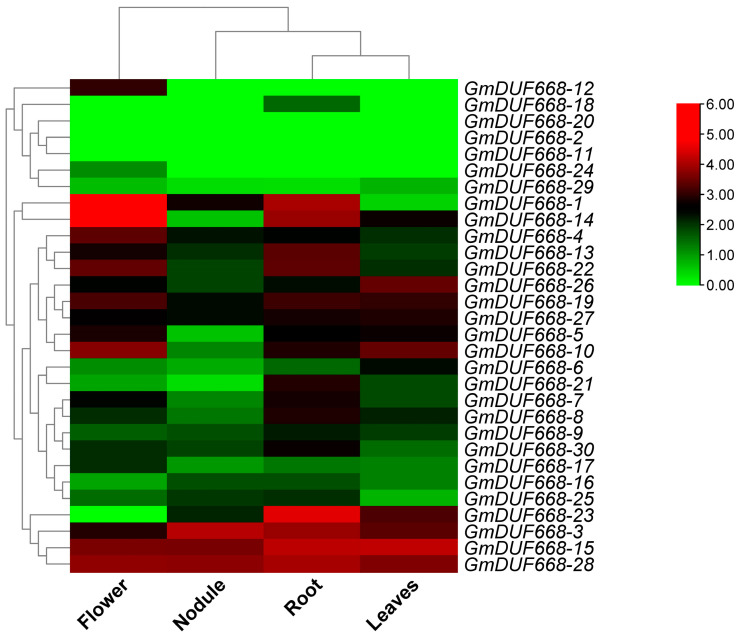
*GmDUF668* expression observed in different tissues. The green, black, and red colors show low to high expression levels.

The expression of 30 *GmDUF668* genes was observed in different tissues (roots, nodules, flowers, and leaves) through the transcriptome dataset (Supplementary Table S6a). The expression heatmap clearly indicated the significant expression of several genes in specific tissues (Figure 7; Supplementary Table S6a). For instance, genes including *GmDUF668-3*, *GmDUF668-4*, *GmDUF668-7*, *GmDUF668-8*, *GmDUF668-9*, *GmDUF668-10*, *GmDUF668-13*, *GmDUF668-15*, *GmDUF668-16*, *GmDUF668-17*, *GmDUF668-19*, *GmDUF668-22*, *GmDUF668-26*, *GmDUF668-27*, *GmDUF668-28*, and *GmDUF668-30* illustrated up-regulation in all examined tissues (root, nodule, leaves, and flowers) (Figure 7; Supplementary Table S6a). However, a few genes such as *GmDUF668-1* and *GmDUF668-25* illustrated up-regulation in the root, nodule, and flower, while *GmDUF668-5*, *GmDUF668-6*, *GmDUF668-14*, and *GmDUF668-21* up-regulated in the roots, leaves, and flower; *GmDUF668-12* and *GmDUF668-24* up-regulated in the flower and *GmDUF668-18* up-regulated in roots (Figure 7). Further results showed that *GmDUF668-2*, *GmDUF668-11*, *GmDUF668-20*, *GmDUF668-24*, and *GmDUF668-29* genes were not up-regulated in any tissue. Furthermore, a few genes also illustrated modest expression responses in different tissues. Overall, the up-regulation patterns illustrated that some specific genes might be significantly involved in soybean development and growth.

### 3.8. GmDUF668 Genes Expression Profiling under Salt Treatment

In this analysis, we investigated the expression of *GmDUF668* genes in response to salt stress using a pre-existing transcriptome dataset (Supplementary Table S6b). Similar to tissue-specific expression, *GmDUF668* genes illustrated differential expression under salt stress. For instance, *GmDUF668-8* and *GmDUF668-20* genes were up-regulated under salt stress at 12 h, while these genes were not up-regulated at 1 h in response to salt stress. Likewise, the *GmDUF668-30* gene also showed up-regulation under salt stress at 12 h compared to 0 h (Supplementary Table S6b). The gene with elevated expression could be genetically engineered to enhance the tolerance under abiotic stress.

### 3.9. qRT-PCR Analysis of GmDUF668 Genes under Salt Stress

**Figure 8:**
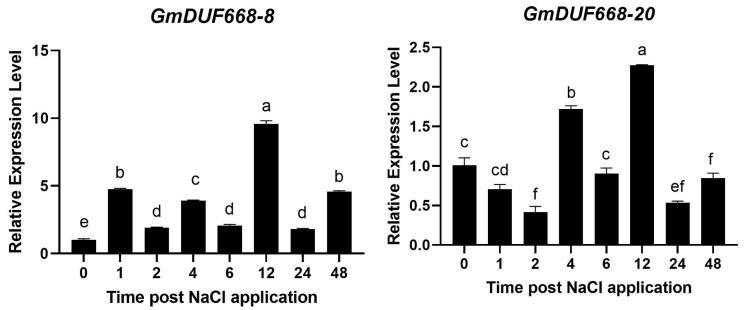
*GmDUF668-8* and *GmDUF668-20* expression under salt treatment using qRT-PCR for 0, 1, 2, 4, 6, 12, 24, and 48 h. For internal control, soybean *Actin* was used. The *x*-axis shows treatment duration, and relative expression levels are depicted by the *y*-axis (a–f).

For expression profiling analysis using qRT-PCR, we chose two *GmDUF668* genes to confirm their transcription levels against salt treatments at different time points (Figure 8). Under salt stress treatment, both of the analyzed genes illustrated higher expression at different time points compared to the control (0 h). The qRT-PCR results showed that *GmDUF668-8* and *GmDUF668-20* genes demonstrated comparatively higher expression levels at 12 h compared to 0 h (Figure 8). Briefly, the selected genes showed similar expression patterns (upregulated) confirmed by the transcriptome dataset mentioned in Supplementary Table S6b.

## 4. Discussion

Soybean (*Glycine max* L.), a member of the *Papilionoideae* family, is a source of edible oil and vegetable proteins [39]. However, soybean production is threatened by environmental stresses, including drought, osmosis, and salinity [40]. The plants have developed a variety of complicated signal transduction routes and signaling systems to protect against cellular damage due to unfavorable environments [41]. The *DUFs* (domain of unknown functions) families contain certain proteins with unknown functions that play a vital role against plant stress. The soybean genome sequences provide the foundation for comprehensive gene function analysis. This study provides knowledge on the genomics of soybean, which helps in *DUF668* gene identification and phylogenetic relations. The identification and function of *DUF668s* were not yet investigated in the soybean. The identification of thirty *GmDUF668s* in the current research illustrated significant roles of *DUF668s* in response to salt stress in soybeans. The occurrence of gene duplication in potatoes has been previously reported for *DUF* genes [6], and the current study’s findings indicate that *GmDUFs* also underwent segmental duplication. Consequently, these results demonstrated that duplication events might be crucial during *GmDUF* gene evolution. Several studies reported the clustering of the *DUF* genes in three different groups. In the current analysis, all *DUF* gene members from different plant species were categorized into three groups. This finding is supported by the phylogenetic study of *A. thaliana*, where all *DUF* genes were classified into three groups [42].

Additionally, the analysis of the gene structure revealed a consistent pattern among *GmDUF668* genes within the same group, as they exhibited similar intron-exon structures. The present study results showed that *GmDUF668* members in the same groups have comparable intron-exon structure and conserved motifs, probably associated with similar tasks under different abiotic stresses. Moreover, in this study, we found the exons ranged from 1–12 while introns from 0–11. A similar gene structure model was observed in previous research, where the exon numbers ranged from 1–12 in *Gossypium hirsutum DUF668* genes [7]. To understand the function of *GmDUF668s cis*-elements in various environmental stress factors, we examined the *GmDUF668s* promoters. We focused on investigating three types of *cis*-elements, including phytohormones, development and growth, and abiotic stress. The participation of the phytohormone and abiotic stress-associated, *cis*-regulatory particles in plant defense was discovered in previous research [7]. Zaynab et al. (2021) reported the role of *cis*-regulatory particles against various forms of environmental stress [43]. The *TaGAPC1* expression pattern in wheat was regulated against salinity and osmotic stress. The *TaGAPC1* promoter contains stress-responsive elements, including LTR, DRE, GTI, and MBS. In addition, our results indicated that *GmDUF668s cis*-particles were involved in plant phytohormones and abiotic stress responses (LTR, DRE, GTI, and MBS). Notably, our research identified the presence of ABA (abscisic acid), MEJA (methyl jasmonate), and SA (salicylic acid) responsive elements, while several studies reported that SA, MeJA, and ABA were associated with plant resistance [44,45,46,47]. These *cis*-acting particles were found in *GmDUF668* genes participating in immunity against stresses. The miRNAs (microRNAs) are ncRNAs (noncoding RNAs) developed from the individual-strand hairpin RNAs predecessor. These showed expressions by attaching a corresponding gene sequence surrounded by targeting mRNAs [48,49]. Several studies reported that miRNAs were involved in different stress and developmental activities [50]. The miRNA156 regulates the anthocyanin biogenesis genes during drought conditions [51]. One study reported that miR167 regulates reproduction in *A. thaliana*. Moreover, the miR167 works as a parental gene that mainly functions with *ARF8* and *ARF6* genes during seed growth [52]. Several miRNAs were observed to participate in the development and stress tolerance processes. For instance, miRNA159 targets grapevine *MYB* genes. The outcomes reported that miRNA159c-*VvGAMYB* significantly participated in grapevine parthenocarpy [53]. The miR395 in transgenic *B. napus* was responsible for lower oxidative stress than in the wild-type. On the other hand, miR159 and miR169 have been found to enhance abiotic stress tolerance, specifically salinity and drought tolerance, in plants [54].

In this research, we observed *GmDUF668s* expression in different tissues using publicly available transcriptome data. Several *GmDUF668* genes represented higher expression levels, mainly in roots, nodules, leaves, and flowers. In potatoes, RNA sequence analysis data were utilized to observe the expression patterns of *DUF* genes in various tissues [43,55]. The results showed higher expression in different tissues. During different developmental stages of tomatoes, the expression of DUF genes was examined, revealing noteworthy findings. Notably, the expression levels were considerably higher in flowers, fruits, leaves, and roots [56]. Yang et al. (2020) investigated *DUF* gene expression in *A. thaliana* and concluded that *DUF* genes display higher expression in different tissues [42]. Similarly, *DUF668* gene expression was observed in rice tissues, illustrating tissue-specific expression patterns [57]. It was concluded that the *GmDUF668* genes’ higher expression in tissues has significant roles in soybean development and growth. Moreover, this study investigated the expression of *GmDUF668s* against salt stress. The findings revealed that *GmDUF668* genes significantly respond to salt stress. The RNA-seq and qRT-PCR results indicated that *GmDUF668-8* and *GmDUF599-20* possessed high expression against salt stress. These outcomes are consistent with earlier reports [58], where *DUF* gene expression was enhanced against salt stress. Furthermore, a study on *A. thaliana* utilized qRT-PCR to observe the expression of *DUF* genes in response to salt stress [42]. The findings illustrated that the *DUF* genes exhibited significant expression against salt stress, indicating their involvement in plant tolerance against abiotic stress. Similarly, in soybean, the expression of *DUF* genes was upregulated in response to abiotic stress factors [59]. During salt stress, the *DUF* genes were mostly highly expressed and upregulated in wheat [58]. These previous findings support current study results and indicate that several *DUF* genes are vital during abiotic stress conditions.

## 5. Conclusions

In this study, we identified 30 *DUF668* genes from the soybean genome. These 30 genes were located on 15 different chromosomes of the soybean genome. To gain a comprehensive understanding of these genes, we performed various analyses, including gene structure visualization, miRNA prediction, and analysis of expression profiling in different tissues and under abiotic stress. Our findings revealed that identified *GmDUF668* genes were classified into three groups. The *GmDUF668* gene expression profiles illustrated that *GmDUF668-8*, *GmDUF668-20*, and *GmDUF668-30* genes play a vital role against salt stress. This study offered a novel understanding of *GmDUF668s* in response to stress resistance and provided foundations for breeding applications.

## Figures and Tables

**Table 1:** Physicochemical features of *GmDUF668s*.

Gene Name	Chromosome	Renamed	Strand	Start (bp)	End (bp)	AA	M.W	PI	Subcellular Localization	Exons
*Glyma.01G101100*	Chr01	*GmDUF668-1*	1	33,585,701	33,587,767	462	52,697.44	8.72	Nuclear	1
*Glyma.03G067800*	Chr03	*GmDUF668-2*	−1	14,004,704	14,006,681	475	54,089.11	8.87	Mitochondrial	1
*Glyma.03G190000*	Chr03	*GmDUF668-3*	−1	40,080,040	40,082,648	570	64,849.72	9.24	Nuclear	1
*Glyma.04G206400*	Chr04	*GmDUF668-4*	−1	47,892,936	47,902,098	631	71,040.11	8.38	Cytoplasmic	12
*Glyma.04G237100*	Chr04	*GmDUF668-5*	−1	50,568,067	50,572,435	607	68,125.75	9.24	PlasmaMembrane	1
*Glyma.04G023800*	Chr04	*GmDUF668-6*	−1	1,905,904	1,911,083	604	67,666.08	9.25	Nuclear	12
*Glyma.05G196700*	Chr05	*GmDUF668-7*	1	38,092,324	3,810,0286	623	69,433.97	9.08	Nuclear	12
*Glyma.06G024000*	Chr06	*GmDUF668-8*	−1	1,800,214	1,805,307	605	68,139.53	9.15	Nuclear	12
*Glyma.06G159200*	Chr06	*GmDUF668-9*	1	13,109,026	13,118,370	628	70,573.44	7.8	Mitochondrial	12
*Glyma.06G127100*	Chr06	*GmDUF668-10*	1	10,419,701	10,425,340	608	68,361.03	9.15	PlasmaMembrane	1
*Glyma.06G159300*	Chr06	*GmDUF668-11*	1	13,124,669	13,132,773	527	59,465.12	8.37	Cytoplasmic	12
*Glyma.07G135700*	Chr07	*GmDUF668-12*	−1	16,070,773	16,072,197	474	54,628.53	9.35	Mitochondrial	1
*Glyma.08G004300*	Chr08	*GmDUF668-13*	1	322,500	330,712	622	69,457.87	8.95	Nuclear	12
*Glyma.09G152900*	Chr09	*GmDUF668-14*	−1	37,537,651	37,538,958	435	48,920.2	8.7	Extracellular	1
*Glyma.10G008200*	Chr10	*GmDUF668-15*	1	787,277	790,619	602	67,449.91	6.91	Nuclear	1
*Glyma.10G178300*	Chr10	*GmDUF668-16*	1	41,158,326	41,160,824	471	53,018.22	8.56	Cytoplasmic	1
*Glyma.10G188300*	Chr10	*GmDUF668-17*	1	42,133,180	42,135,536	589	66,288.73	7.6	Nuclear	1
*Glyma.10G065400*	Chr10	*GmDUF668-18*	−1	6,242,662	6,245,760	564	64,573.75	9.59	Mitochondrial	1
*Glyma.10G218000*	Chr10	*GmDUF668-19*	−1	44,991,655	45,002,480	659	73,611.29	7.38	Nuclear	12
*Glyma.10G201200*	Chr10	*GmDUF668-20*	1	43,234,472	43,238,381	600	67,097.61	9.37	Mitochondrial	1
*Glyma.14G219500*	Chr14	*GmDUF668-21*	1	48,448,248	48,454,092	592	67,337.61	9.21	Nuclear	12
*Glyma.16G203800*	Chr16	*GmDUF668-22*	−1	36,470,848	36,472,257	469	52,736.92	9.37	Mitochondrial	1
*Glyma.17G258400*	Chr17	*GmDUF668-23*	1	41,248,979	41,254,225	593	67,203.75	9.35	Nuclear	12
*Glyma.18G185600*	Chr18	*GmDUF668-24*	−1	44,608,314	44,609,744	462	52,611.9	9	Mitochondrial	2
*Glyma.19G190300*	Chr19	*GmDUF668-25*	−1	44,794,562	44,797,054	577	65,532.4	9.39	Nuclear	1
*Glyma.20G189200*	Chr20	*GmDUF668-26*	−1	42,780,095	42,784,113	600	67,446.87	9.31	Mitochondrial	1
*Glyma.20G174000*	Chr20	*GmDUF668-27*	1	41,143,103	41,155,330	654	72,588.1	8.69	Nuclear	12
*Glyma.20G087700*	Chr20	*GmDUF668-28*	−1	32,765,127	32,768,858	603	67,963.67	8.4	Nuclear	1
*Glyma.20G202500*	Chr20	*GmDUF668-29*	−1	43,951,663	43,953,439	543	61,301.29	6.77	Nuclear	3
*Glyma.20G211800*	Chr20	*GmDUF668-30*	−1	44,844,951	44,847,383	473	53,088.22	8.88	Mitochondrial	1

**Table 2:** Duplicated *GmDUF668* gene pairs and their expected divergence times.

Seq_1	Seq_2	Ka	Ks	Ka_Ks	Selection Pressure	t (MYA)	Duplication Type
*GmDUF668-19*	*GmDUF668-27*	0.02097806	0.10881538	0.1927858	Purifying	8.83094206	Segmental
*GmDUF668-12*	*GmDUF668-24*	0.068095042	0.277752741	0.24516425	Purifying	22.54109994	Segmental
*GmDUF668-14*	*GmDUF668-22*	0.035564017	0.167503706	0.212317791	Purifying	13.59380923	Segmental
*GmDUF668-17*	*GmDUF668-29*	0.023718981	0.161984454	0.146427512	Purifying	13.1458928	Segmental

## Data Availability

The protein sequences of Arabidopsis and other species data were downloaded from the Phytozome database (https://phytozome.jgi.doe.gov/pz/portal.html (accessed on 11 December 2022). The original contributions presented in this study are included in the article/Supplementary Materials, further inquiries can be directed to the corresponding authors.

## Supplementary Materials

The following supporting information can be downloaded at: https://www.mdpi.com/article/10.3390/plants12162923/s1, Table S1: The primers used for the qRT-PCR analysis; Table S2:The protein sequences of DUF668 family genes in Glycine max, Arabidopsis thaliana, Brachypodium distachyon, and Sorghum bicolor; Table S3: The information of identified 10 motifs in GmDUF668 proteins; Table S4: Information of hormone- and stress-related cis-elements detected in the promoter regions of GmDUF668; Table S5: Information of predicted miRNA targeting GmDUF668 genes; Table S6a. Transcriptome expression of GmDUF668 genes in different tissues; Table S6b. Transcriptome expression of GmDUF668genes against different stress conditions.
